# The COVID-19 Pandemic from the Health Workers’ Perspective: Between Health Emergency and Personal Crisis

**DOI:** 10.1007/s42087-021-00232-z

**Published:** 2021-06-23

**Authors:** Tiziana Marinaci, Claudia Venuleo, Giulia Savarese

**Affiliations:** 1 Laboratory of Applied Psychology and Intervention (Lab P.A.I), Department of History, Society and Human Studies, University of Salento, Lecce, Italy; 2 Department of Medicine, Surgery and Dentistry “Scuola Medica Salernitana”, University of Salerno, Fisciano, Italy

**Keywords:** COVID-19, Health workers, Narrative inquiry, Meaning

## Abstract

Different scholars have emphasised the psychological distress experienced by health workers during the COVID-19 pandemic; however, there are almost no qualitative studies and we know very little about the everyday experience of this group. The present study’s goal was to explore how health workers interpreted the meaning of the pandemic crisis in their life. An online survey was available during the Italian lockdown. Respondents were asked to write a passage about the meaning of living in the time of COVID-19. A total number of 130 questionnaires (M = 42.35; DS = 10.52; women: 56.2%) were collected. The Automated Method for Content Analysis (ACASM) procedure was applied to the collected texts to detect the factorial dimensions underpinning (dis)similarities in the respondents’ narratives. Such factors were interpreted as the markers of latent dimensions of meanings (DS). The two main DS that emerged were characterised by the pertinentisation of two extremely basic issues: what the pandemic represents (health emergency versus personal crisis) and its impact (powerlessness versus discovery of new meanings). On the whole, health workers’ narratives help to highlight the risk of normalising the feelings of fear and impotence experienced when facing the health emergency and the need to recognise that such feelings are strictly intertwined with the limited resources received to “face the battle”; the need to recognize the human vulnerability of the women and men “inside the lab coat” and the human effort to maintain or reconstruct a sense of self and purpose in the face of troubled circumstances.

## Introduction

The COVID-19 pandemic presents unprecedented psychological threats to the clinician’s well-being and to health care systems (Albott et al., 2020; Lai et al., 2020; Liu et al., 2020; Nickell et al., 2004; Preti et al., 2020; for a review: Spoorthy et al., 2020). Different scholars, in the field of clinical psychology and public health research, emphasised high levels of psychological stress and anxiety, psychosomatic symptoms, and a higher risk of burnout experienced by health workers (Marinaci et al., 2020; Montemurro, 2020; Sasangohar et al., 2020). Similar psychosocial impacts were reported during the AIDS epidemic in the early 1980s, the Severe Acute Respiratory Syndrome (SARS) outbreak of 2003 (Chen et al., 2005; Grace et al., 2005; Lu et al., 2006; Su et al., 2007), the A/H1N1 influenza pandemic (Goulia et al., 2010), and most recently, the Ebola virus epidemic in West Africa in 2014 and 2015 (Lehmann et al., 2016).

In this paper, we argue that, while on the one hand, the overriding focus on the psychological distress of the health emergency is crucial to identifying psychological needs and adequate responses; on the other hand, it imposes a specific perspective on the ways health workers experienced the pandemic and interpreted its meaning in their life: specifically, the view that psychological distress was experienced as a result of facing extraordinary but natural circumstances and that the problems at stake were seen as related to the professional role exercised in those circumstances, more than for example to fear and worries about family’s responsibilities and demands. However, neither of the assumptions can be taken for granted. One can argue that most of the factors cited to explain physical and mental fatigue, stress and anxiety, and burnout among health workers, such as limited resources, longer shifts, disruptions to sleep and to the work-life balance, and occupational hazards (Adams & Walls, 2020), are not intrinsically related to the pandemic; rather, they may reflect political and management choices (Cheong & Jones, 2020; Heymann et al., 2015; Khubchandani et al., 2020; Krystal et al., 2020; Madhav et al., 2017; Phelan et al., 2020). With regard to Italy, the context of the current study, where about 134,116 health-care workers have been infected (Bulletin of the integrated supervision of the Istituto Superiore di Sanità (ISS, Rome) and Istituto Nazionale di Statistica (Istat, Rome), updates 21 May 2020) and 359 have died (National Federation of Surgeons and Dentists Orders (FNOMCEO, Rome), updated 21 May 2020), the COVID-19 health emergency happened within a health system already suffering from a progressive decrease in resources allocated for health-related research and public health. Today, Italy is below the average for both total health expenditure per capita (USD 3428 vs USD 3980) and public expenditure (USD 2545 vs USD 3038), ahead of only the Eastern European countries and Spain, Portugal, and Greece) Organisation for Economic Cooperation and Development (OECD), updated 10 September 2020). Insufficient availability of medical personnel, products, and physical structures (e.g., hospital and community health workers, personal protective equipment and sanitisers, reagents and swabs, ventilators and beds, medications and vaccines) are among the most evident factors which highlighted the low degree of preparedness of Italy (among other many countries) to respond effectively to the COVID-19 pandemic and the paradoxical circumstances of health workers, called on to safeguard people’s health in the absence of physical and psychological protection (Marinaci et al., 2020).

Pressures related to the professional role and workload in hospitals are only one aspect of the issue. Family concerns may constitute another important source of distress. In their study based on focus group interviews conducted with a total of 30 General Surgery residents in 2 Boston centres, He et al. (2020) identify as factors of common concern not only the risk of being infected by patients, but also the risk of spreading infection to family members. Shanafelt et al. (2020), who conducted eight listening sessions with groups of physicians, nurses, advanced practice clinicians, residents, and fellows (involving a total of 69 individuals) during the first week of the COVID-19 pandemic, found that along with inadequate PPE, lack of rapid testing available in the face of symptom development, lack of up-to-date information and communication, and feelings of inadequacy if deployed to new areas, other important sources of distress were the fear of carrying infection to family members, concerns related to the access to child care resources, and the need for support for other personal and family needs.

However, as pointed out by the recent meta-analysis of Kisely et al. (2020), qualitative studies are nearly totally absent in the evaluation of the psychological impact of pandemics on health workers, so we know very little about the everyday experience (feelings, experiences, practices, actions) and perspectives of this group. To our knowledge, no study in Italy has examined the meaning of the pandemic crisis by adopting the health workers’ perspective.

Framed with the socio constructionism epistemology and a semiotic and cultural perspective, this study aims to fill this gap and to exploratively analyse how health workers interpret the meaning of the pandemic crisis in their life.

## The Present Study

### Theoretical Framework

According to social constructionist epistemology (Gergen, 1999; McNamee & Gergen, 2000; Sharf & Vanderford, 2003), the discourses produced on the impact of the pandemic on health workers’ well-being are regarded as one of the multiple structures of intelligibility (Gergen, 1985) that can be selected to depict the meaning of the pandemic crisis for this group. Any inquiry into human nature is itself inevitably a storytelling enterprise in the sense that it is not confined to describing, but establishes what counts as data (Bruner, 1986; Law, 2011). The process of foregrounding some kind of problems more than others is not without implications. As Bacchi (2009) notes, the way a problem is defined, even implicitly, has flow-on effects as to how the policy is imagined. For instance, the predominant focus on the psychological impact of the pandemic on health workers prompts strategies of intervention centred on the individual (typically the need for psychological support) rather than, for example, on the settings and systems within which the disease is manifested and develops. Constructionism asks one to suspend belief that commonly accepted categories or understandings receive their warrant through observation of the “reality” (Gergen, 1985). The definition of what is a problem and what not, how they originate, and how they can be addressed need to be seen as emerging within complex networks of meaning, subjective experience and social relations (Venuleo & Marinaci, 2017; Vrecko, 2010).

Semiotic Cultural Psychological Theory (SCPT) is part of this stream of thought. At the core of the SCPT, there is the view of the individual as a semiotic subject (or sense-maker) (Shweder & Sullivan, 1990), namely, a subject engaged continuously with the interpretation of experience (Salvatore, 2018; Valsiner, 2007; Valsiner et al., 2016; Van Herzele & Aarts, 2013). Each activity of interpretation (i.e., sensemaking) is seen as channelled and constrained by basic, bi-polar dimensions of affect-laden generalised meanings (e.g., pleasant/unpleasant; passivity/engagement) which foster and constrain the way the sense-maker interprets any specific event, object or condition he deals with (Bento, 2013; Russo et al., 2020; Salvatore et al., 2018). Believing that “life is a matter of duties and sacrifices” is an example of a generalised meaning which does not concern a specific aspect of experience but can act as an interpretative category able to guide and constrain people’s way of feeling, performing and talking about their attempt to deal with the health emergency, as well as other situations and problems of their life. Different studies have highlighted how interpretations are not merely abstract judgments, but a way of being channelled to act and react in a certain way, enabling attitudes and behaviour to be oriented (e.g., toward vaccination: Rochira et al., 2019; the otherness: Salvatore et al., 2019; the ways of evaluating a public service: Venuleo, 2013; the assumption of hazardous behaviours: Venuleo et al., 2015).

Accordingly, the value of adopting the health workers’ perspective and thus valorising their personal accounts of the meaning of the pandemic in their life as “data that count” lies in acknowledging the ways they experience, cope, and react to the circumstances and challenges related to the pandemic. Similarly, the ways they evaluate the actions planned to respond to “their needs” depend on how they represent the pandemic crisis, the change it imposed, the needs it brought to the fore, and what should be learnt from it. For instance, in a representation of the pandemic crisis as health emergency fuelled by social-political conditions and institutional inadequacy, it is likely that the support strategies for health workers focusing exclusively on their psychological distress were seen as an inadequate response to the socio-cultural and institutional determinants identified as the source of their distress.

According to SPCT and consistently with other semiotic and cultural perspectives, people’s sensemaking does not develop in a social vacuum; the cultural milieu—as a system of semiotic practices (e.g., discourses, behavioural scripts)—places constraints upon the virtually infinite ways people can make sense of the events, problems, circumstances of their life, and/or the community (Cannon & Müller-Mahn, 2010; Marinaci et al., 2019; Salvatore & Zittoun, 2011).

The analysis of how health workers interpreted the crisis scenario therefore provides insight into the connections between the individual and society, since their interpretative categories are also made up of a repertoire of meanings which prevails in a wider socio-cultural and historical context (Bruner, 1990, 1991; Garro & Mattingly, 2000; Maynes et al., 2012).

### Goals of the Study

The study aimed to investigate the interpretative categories adopted by health workers to interpret the pandemic scenario and its meaning in their life. Since qualitative studies of this kind are nearly totally lacking, no specific hypothesis guided the study. We were interested to exploratively examine their ways of thinking and making sense of the circumstances: what they say about the crisis, the kind of change it imposed, the problems they face, and the needs the crisis brought to the fore.

## Method

An anonymous online survey was available online from 1 April to 19 May 2020, the period when the Italian government imposed self-isolation. The survey was aimed to analyse the impact of the COVID-19 pandemic on health workers, based on a mixed-method. A section of the survey was devoted to analysing the relationship between emotional distress, psychosomatic symptoms, and perceived adequacy of the institutional responses, through data emerging from self-report questionnaires (Marinaci et al., 2020). Another section (the subject of the current study) was devoted to analysing, with a qualitative approach, the health workers’ subjective experience of the pandemic crisis. Narrative inquiry was chosen to this end.

According to the theoretical frame guiding the study, narratives are important not because they furnish an accurate picture of what actually happened but because they show how people understand and live their experience (Gergen, 1985) and because they are ways to participate actively in the practice of a particular culture. Personal narratives can capture attitudes, beliefs, and values about themselves as individuals (Baxen, 2008), their ways of making sense of social experience and of their own role in it, and mirroring the changing social conditions (Bertaux, 1981) and the place of individuals within them (Andrews, 2007). They also serve to dispute misleading generalizations and universal claims, and to provide unique insight into the connections between individual and society, since they take place within the context of ongoing social debates and are shaped in part by a speaker’s awareness of and response to these, in terms of anticipated disagreements and counter-arguments (Billig, 1987; Taylor, 2006).

The health workers’ subjective experience was investigated through the request to write about the meaning of “living in the time of COVID-19…” The open text was purposely chosen to allow the participants to write down everything that came to mind about the situation. They were encouraged to respond in the manner deemed most appropriate, taking into account that the objective of the investigation was to collect their subjective experience, and to take the space they needed. Unfortunately, we do not ask to describe the circumstances under which the survey was filled out (e.g., home, work), a setting condition that could have had an impact on the way of relating to the proposed stimuli.

### Participants

The health workers were recruited on a voluntary basis following a snowball sampling methodology. Accordingly, a small pool of initial informants (physicians, nurses, and social care workers) was asked to share the link to the survey through their social networks with other frontline health workers who could potentially contribute to the study.

A total number of 130 questionnaires and related texts (mean age = 42.35; DS = 10.52; women: 56.2%) were collected. The texts varied in length from half a page to a page and a half and differ with regard the balance between reflection and more straightforward reporting of events and the degree of comments related to their medical role and to their more private sphere; a heterogeneity which appears consistent with the plurality of the ways health-workers can interpret the shared experience.

Regarding their professional activity, 74.4% were front-line health workers (physicians, nurses, general practitioners, surgeons, social-health operators), the other professionals working in a hospital as psychologists, psychiatrists, lab-technicians, or administrative staff.

### Data Analysis

An Automatic Procedure for Content Analysis (ACASM; Salvatore et al., 2012, 2017a, b)—performed by software T-Lab Plus 2020 (Lancia, 2020)—was applied to the whole corpus of narrative texts collected. ACASM is a member of the broader family of methods of semantic analysis focusing on the co-occurrence of lexical units (e.g., ALCESTE, cf. Reinert, 1993; latent semantic analysis, cf. Landauer & Dumais, 1997). Compared to most such methods, the main specificity of ACASM is that it adopts a single sentence or group of a few sentences as its unit of context (the unit of context is the segment of text within which co-occurrences are detected). ACASM chooses this unit of context in order to make the semantic analysis sensitive to the contextual nature of linguistic meaning: the meaning of any word is not fixed but depends on the way it combines with other words in the contingent dynamics of talk. The analysis is designed to map the meaning variability—the set of different themes (i.e., beliefs; opinions; combined statements; verbal images) emerging in response to the open stimulus—*structurally*, namely, to capture the main *dimensions of meaning* underpinning and giving sense to the different set of contents characterizing the texts. Each dimension can be conceived of as a component/quality of the subject which was made pertinent by the participants and provides space for a plurality of statements and positions. For instance, insofar as the participants make pertinent the impact of the pandemic on their daily life, then this semantic component provides space to express different statements/connotations on this aspect (e.g., one participant might say “life changed totally” whereas another might say “after the first little while everything returned to normal”). Thus, the structural map of the meaning variability goes beyond the descriptive level of the content analysis and identifies the semantic structure generating the variability of the contents. (Salvatore et al., 2017a, b; Visetti & Cadiot, 2002).

ACASM procedure followed two steps. Firstly, the textual corpus of narratives was split into units of analysis, called Elementary Context Units (ECUs) and the lexical forms present in the ECUs categorized according to the “lemma” they belong to. A lemma is the citation form (namely, the headword) used in a language dictionary: for example, word forms such as “child” and “children” have “child” as their lemma. A digital matrix of the corpus was defined, having as rows the ECU, as columns the lemmas and in the cell x_*ij*_ the value “1” if the *j*th lemma was contained in the *i*th ECU; otherwise, the x_*ij*_ cell received the value “0.” Table 1 describes the characteristics of the dataset.

**Table 1:** Dataset

	*N*
Texts in the corpus	130
Elementary contexts (EC)	214
Types	2116
Lemma	798
Occurrences (Tokens)	7311
Threshold of lemma selection	3
Lemmas in analysis	293

Secondly, a Lexical Correspondence Analysis (LCA)—a factor analysis procedure for nominal data (Benzécri, 1973)—was carried out on the matrix obtained, to retrieve the factors describing lemmas with higher degrees of association, i.e., occurring together many times. Each factorial dimension describes the juxtaposition of two patterns of strongly associated (co-occurring) lemmas and can be interpreted as a marker of a latent dimension of meanings underpinning dis/similarities in the respondents’ narratives (Salvatore et al., 2017a, b). The interpretation of the factorial dimensions is carried out in terms of inferential reconstruction of the global meaning envisaged by the set of co-occurring lemmas associated with each polarity. The first two factors extracted from LCA were selected as the ones explaining the broader part of the data matrix’s inertia, and labelled by three experienced researchers, in double-blind procedure. Disagreement among researchers was overcome using a consensus procedure (Hill et al., 1997).

The LCA provides a measure of the degree of association of each respondent with every factorial dimension, expressed in terms of the respondent’s position (coordinate) on the factorial dimension. Once the coordinates of each subject were identified, an ANOVA with post hoc analysis based on the Bonferroni test was computed to examine potential (dis)similarities related to gender and professional role.

## Results

### Dimensions of Meanings

Tables 2 and 3 illustrate respectively the first and the second factorial dimensions obtained by the ACASM procedure. For each polarity of the two dimensions, the lemmas with the highest level of association (V-Test) are reported, as well as their interpretation in terms of labelling of their meaning. Henceforth, we adopt capital letters for labelling the dimensions of meaning and italics for the interpretation of polarities.

**Table 2:** LCA output. First factorial dimension

Representation of the pandemic crisis			
Health crisis ( −)		Personal crisis ( +)	
Lemmas	Test value*	Lemmas	Test value*
To activate	−4127	Everyday life	11,641
Mask	−4069	Change	7741
Protection	−3756	To rediscover	6383
Expense	−3323	Importance	5465
Glove	−3315	Period	5262
China	−3257	Anxiety	4897
Nurse	−3003	Bewilderment	4742
To wear	−2984	Inadequacy	4713
Virus	−2968	Important	4443
Intensive care	−2692	Tragic	442
Contagious	−2688	Single	4336
To infect	−2633	Suspended	4271
Epidemic	−2605	To face	4
To continue	−2555	Loneliness	3719
To close	−2549	One time	3713
Physician	−2524	To cultivate	3667
To begin	−2522	Fear	3642
To need	−2521	Sense	3245
Month	−2521	Habit	3205
Close	−2512	Affect	3184

^*^Highest levels of association standard scores (V-Test)

**Table 3:** LCA output. Second factorial dimension

Impact of the pandemic crisis			
Powerlessness ( −)		Discovery of new meanings ( +)	
Lemmas	Test value*	Lemmas	Test value*
Anxiety	−5208	Everyday life	12,535
Limitation	−4683	To rediscover	6893
Sense	−4463	Tragic	6671
Fear	−4445	Importance	6398
Stress	−4267	Single	4672
To permit	−4164	Little	4668
Lived	−4115	Weak	4545
To clean	−3858	Change	4323
Sadness	−3828	China	4266
To live	−3828	At the beginning	3338
Reaction	−3811	Important	329
Period	−3652	First	3272
Uncertainity	−3622	Epidemic	322
Glove	−3267	Italy	3137
Respect	−2881	To understand	2953
For me	−288	Sky	2859
Service	−2842	School	2717
Inadequacy	−2751	Partner	2704
One time	−2723	Force	2688
Management	−2717	To cause	2615

^*^Highest levels of association standard scores (V-Test)

FIRST FACTORIAL DIMENSION. REPRESENTATION OF THE PANDEMIC CRISIS: *health crisis* versus *personal crisis.*

This dimension opposes two patterns of lemma which we interpret as the markers of two ways of representing the pandemic crisis (Table 2).

(-) Health crisis. On the negative polarity, discourses focus on the health emergency (*virus*, *China*, *contagious*, *epidemic*, *to infect*), its impact on the health system (*nurse*, *physician*, *intensive care*), and the devices and measures to contain the spread of the contagion (*protection*, *to wear*, *mask*, *glove*, *gel*, *closed*, *to close*). The health workers talk about the invisible and unannounced enemy which forced people to self-isolate, terrifying because it was unknown and unpredictable, claiming victims and spreading fear and anguish, in the face of which one can only attempt to contain the damage. The lack of knowledge on how to eradicate it and the condition of having to act by trial and error are emphasized as a source of feelings of impotence and uncertainty “outside time” (“It seemed like it would never pass”).

*Anguish. It was a shocking and unimaginable experience, every day seeing the one-sided struggle that was waged against Covid-19 without real knowledge, without a proven cure and with the death toll always rising, with fear for loved ones, for the future of the children, hostages of a virus that claimed victims and spread fear *(Man, front-line)

*We went through a long period of uncertainty, where the knowledge we had was too little and we advanced by trial and error, trying to limit the damage. It seemed like it would never pass and that every day was worse than the last. But only those who had more or less direct contact with the virus understood the tragedy* (Woman, front-line)

*As this Covid unfortunately was not known we did not have a therapy to defeat it or even the vaccine.* (Woman, front-line)

At the same time, the initial underestimation of the problem by the experts, claiming that the problem concerned someone on the other side of the globe, and the health system’s lack of preparedness to wage the “war” are foregrounded:

*It all started after New Year’s Eve 2020. From China came the news that a new virus, highly infectious, was in circulation. To begin with, the idea that China was on the other side of the planet gave everyone the feeling that we couldn’t care less about the problem. The experts rushed to say, 'The virus will never come to us'. But no! Within a few weeks we found ourselves in Italy, which had the record of being the first country, after China, to be affected by what would turn out to be an epochal epidemic. *(Man, front-line)

In the health-workers’ narratives, many weaknesses of the health systems are listed: the lack of information and guidelines, the paucity of human resources and the overwork, and the lack of individual protection equipment the inadequacy of instruments to care for the patients. On the whole, the co-occurrence of discourses focused on the unpredictable character of the virus and of discourses on the lack of preparedness of the health systems suggest the view that institutional responses played a significant role in increasing the risk (of being infected and of infecting others) and the feeling of being unprepared and helpless.

*To date, we have 5 Covid departments with recovery of professionals from surgery and outpatient areas. PPE was only made available in Covid wards and no PPE was provided in the rest of the wards, which would result in a non-containment of the spread and the onset of contagion between health-worker patients and staff.* (Woman, no front-line)

*Covid 19 determined health emergency (there was a shortage of Doctors, nurses, staff etc. Also a lack of ventilators and masks etc.)* (Woman, front-line)

*I felt the enormous gravity of this health emergency and the need to take action in every way to protect myself, other staff and patients from possible contagion, aware of the lack of information and therapies from the scientific world. I experienced the shock of the lack of gloves, masks, clean gowns, wipes and disinfectant gel. *(Woman, front-line)

( +) Personal crisis*.* On the positive polarity, lemmas relate to the disruptive changes that occurred in daily life (*change*, *tragic*, *everyday life*, *habits*) and their impact on a personal level: negative affect (*anxiety*, *fear*) and the feeling of inadequacy (*inadequacy*, *bewilderment*) faced alone *(single*, *loneliness*, *to face*) co-occur with a process of discovery of new meanings and priorities (*to rediscover*, *sense*, *importance*, *important*, *affect*). Two sides of the crisis are recounted. On the one hand, the loss of what the pandemic has no longer made possible and has interrupted in one’s daily life: leisure time but above all, the closeness to family and friends, the gestures of love, and the possibility of taking care of the people who count in their life. The present is felt to be a nightmare, characterized by feelings of anguish related to the physical distancing measures and fear for their own health and that of their loved ones, a fear fomented by media communication, dominated by the focus on the health emergency, and the daily toll of infections and deaths, like a war bulletin. The feeling of impotence finds relief only in the hope that a medical antidote is identified; it is significant that someone defines the antidote as a miracle, by definition something that can reverse the course of events (bringing back the pre-rupture life) and which refers to a supernatural intervention, in that it exceeds the limits of the normal predictability of events or goes beyond the possibilities of human action.

*Suddenly, in a moment, everything stopped. Everything that at that time seemed indispensable to me, disappeared (trips, gym, walks, dinners with friends (...). The television, which was supposed to be a source of entertainment, was continuously informing about the emergency situation that we were experiencing: the overflowing intensive care units, the number of infections and hundreds of deaths every day. It was like watching a war bulletin. Fear dominated, and there were sleepless nights. The only relief, in that disastrous situation, was the hope that some scientist could find the miracle cure, vaccine or therapy, so as to put an end to the pandemic.* (Woman, front-line)

*There was concern about a possible contagion, not so much for my own health, but for that of the people close to me. (...) I haven’t seen the family for almost two months, and I’ve missed them. I never wanted to go home for fear of taking the virus with me. The most painful thing of all this time was the diagnosis of cancer that my mother received. The virus kept me from being there for her, comforting her in person, embracing her, while she cried.* (Man, front-line)

*The anguish, the astonishment, the disorientation, the fear.* (Man, front-line)

On the other hand, the health-workers speak about how the negative feelings related to the pandemic crisis and the rupture of their daily life provided special opportunities to reconsider the value of what is normally taken for granted: e.g., the sounds of nature, the value of the affects, and taking care of oneself. It is worth noting that—based on the health workers’ narratives—the possibility of reacting to and coping with negative affect and events depends on the individual’s capacity to turn the crisis into opportunity, more than on the confidence that changes might occur in the circumstances which have fuelled fear, impotence and uncertainty.

*Living in the time of the covid meant fear, impotence, waiting, an uncertain future, breathlessness, anxiety, insomnia, fear of others and of oneself for others, lack of affection and gestures of love, unhappiness, but at the same time rediscovery of the sounds of nature, especially silence and rediscovery and the possibility of cultivating domestic life and home affections, and time to cultivate certain passions and take better care of oneself and in particular to realize the importance of some relationships and their manifestations of love, often not considered in normal everyday life*.(Woman, front-line)

*I went through a period of existential uncertainty, of sadness, for a person who loves contact with others... Of fear, of precariousness, but also of resilience. Of strengthening the authentic bonds, and of contact with myself. *(Woman, no front-line)

*An eternal day zero. In which the present is a closed box without a window on tomorrow. The lack of planning for the future that is too uncertain and that forces us to appreciate every day*. (Woman, front-line)

SECOND FACTORIAL DIMENSION. IMPACT OF THE PANDEMIC CRISIS: *powerlessness* versus *discovery of new meanings.*

This dimension contrasts two patterns of lemma which we interpret as the markers of two ways of representing the impact of the pandemic crisis (Table 3):

(-) Powerlessness. Lemmas which refer to the actual or potential measures to respond to the health emergency (*limitation*, *to allow*, *to clean*, *glove*, *period*) co-occur with lemmas which refer to the constraints in the managing of the health system (*services*, *management*, *inadequacy*) and to the experience (*lived*, *to live*) of psychological distress (*reaction*, *anxiety*, *fear*, *stress*), feeling of *uncertainty*, and of not being respected in their own work (*respect*, *for me*, *sadness*). The narratives focus on the strength to take all possible measures to protect themselves and others (family members, colleagues) from infection and the dismay at experiencing the lack of the personal protective equipment necessary for this purpose. From this perspective, the anguish is not told as the mere reaction to a natural hazard, but as the by-product of a perceived gap between one’s own feeling (the severity of the situation, the need to take all possible precautions) and the tools actually available.

*I felt the enormous gravity of this health emergency and the need to take action in every way to protect myself, other staff-members and patients from possible infection, aware of the lack of information and support from the scientific world. I experienced the dismay of the lack of gloves, masks, clean shirts, wipes and disinfectant gel*. (Woman, front-line)

*Powerlessness, great care in keeping a distance from people, frequent washing of hands that has ruined my skin, a lot of anguish for me and my family about possible infections*. (Woman, no front-line)

A widespread feeling of impotence marks the narratives: impotence related to the difficulty in treating patients, associated to feeling unprepared and inadequate, despite enormous efforts; impotence toward people who die in their home without assistance; impotence toward the colleagues falling ill in the course of their work; and impotence, above all, related to the feeling of being left alone in the middle of a tsunami: left alone by ordinary people and their poor compliance with the protective behaviours necessary to contain the spread and left alone by the institutions and the inadequacy of health management.

*Incompetence of the institution and ignorance of the masses*. (Man, front-line)

*Anxiety, so much apprehension, bewilderment, fear of a radical change in our habits. Confirmation of the inadequacy of our administrators*. (Man, no front line)

*As a health care worker, the impression of being in the middle of a tsunami where it was difficult to see the right way to go in the crowding and chaos of the departments, the emergency room... Finding out with horror that some sick people died at home alone without assistance! Anger because some areas should have been closed down earlier regardless of economic interests. Appreciation for the great ability of colleagues to get involved and to be present in the field of battle, to know how to reorganize the life of a large hospital to give the best assistance to the sick in this new plague of the 2000s. Regret at seeing the cancellation of all the initiatives planned to improve the stay of child cancer patients in hospital, now all day confined to a room with their parent*.(Woman, front-line)

*It was like fighting a war...against a terrible, invisible enemy. It made us feel incompetent, inadequate and often alone. I am the coordinator of a facility for the elderly and the pain that was experienced in this period was immense...for guests who were sick and left us, for operators who were sick in turn, for family members who could not be close to their loved ones... the pain was really too much, all at once. We won’t be the same people ever again*. (Woman, no front-line)

( +) Discovery of new meanings*.* Lemmas which refer to the first countries hit by the health emergency (*China*, *Italy*, *epidemic*, *tragic*) and temporal markers (*at the beginning*, *first*, *week)* seem to depict the initial moment of the pandemic, with the sudden changes that occurred in daily life (*to cause*, *change*, *everyday life*, *school*, *companion*). The rupture of habits is here associated to the rupture of well-established certainties and the personal discovery of new meanings and priorities (*single*, *to rediscovery*, *to understand*, *weak*, *little*, *importance*, *important*); a kind of turning point which highlights the fragility of human life but, at the same time, calls for the need to understand what is trivial and what matters. Most discourses focus on the different perspective acquired towards the “little things” of daily life, previously taken for granted, and toward one’s goals and personal priorities: greater awareness of one’s emotions and more attention toward family relationships are the main outcomes cited of the changes that occurred in the private sphere.

*I felt life flow over me in its precarious beauty, that makes you think of how precious the pomegranate is on the terrace with its new buds and the early morning blue sky and the Easter egg that you bought for your daughter anyway even though she is a thousand miles away and "then we’ll eat it when you come back", so I’m sure she’ll come back and I’ll hold her like when she was little even though she’s 26*.(Woman, front-line)

*Growth, a suspended time in which you can take care of yourself, the most important family relationships. A period in which one has to acquire a great awareness of oneself and of one’s own emotions, especially fear and anxiety, sometimes so intense. A period in which to think back to oneself, to the scale of one’s goals and the way to go forward. A time to weigh things up*. (Woman, no front-line)

*A difficult time, which was also a healthy shake-up of the whole framework of useless things that filled our lives*. (Man, front-line)

Other discourses more clearly depict the pandemic crisis as a turning point which potentially invests both the private sphere and the social one, undermining the idea of invincibility of human beings, calling for a reorganisation of the social-political systems, and highlighting the importance of science, research, and health policies in daily life. The pandemic crisis—the discourses suggest—highlighted how closely intertwined are the individual level of the personal psycho-physical health and the systemic level of the governance of society’s economic and health conditions.

*Covid-19 made us get off a moving train, and face reality, that we are not invincible; it reminded us to appreciate more the small everyday things that we took for granted. Covid-19 unmasked us, revealing what were the real priorities for each of us*. (Woman, no front-line)

*Time for reflection and personal growth. So much solitude and desire to change the system. There is so much to do*. (Man, front-line)

*A special and unexpected period that has allowed us to implement our working and personal skills and stressed the importance of science, research and health policies in daily life*. (Woman, front-line)

*At the beginning, a feeling of discomfort and constraint, then with the passing of the weeks, a rediscovery of time, of the family, of reading, of one’s own self, in a word, of freedom in its broadest sense* (Man, front-line)

## Relation Between Dimensions of Meaning and Socio-demographic Characteristics

Significant differences among front-line/non front-line health workers were found on the first dimension extracted, that is “Representation of the pandemic crisis” (F_f1_(1, 121) = 4.225; p < 0.05). Post hoc analyses using the Bonferroni test showed that front-line health workers score lower than non front-line (Mean Difference _I – J_ =  −0.3677; *p* < 0.01); namely, it is the front line that mainly interprets the pandemic in terms of health emergency (negative polarity). No significant differences were found related to gender.

## Discussion

The first goal of the study was to explore the Dimensions of meanings (DS) through which Italian health workers represented the pandemic crisis and its meaning in their life. The analysis of the narratives based on the ACASM procedure led to the identification of two main dimensions of meaning which foreground two very basic issues: what the pandemic crisis represents (HEALTH EMERGENCY versus PERSONAL CRISIS) and its impact (POWERLESSNESS VERSUS DISCOVERY OF NEW MEANINGS).

Specifically, along the first dimension (Representation of the pandemic crisis), the health crisis polarity brings to the foreground the health emergency and the devices and measures to contain infections and to avoid the overloading of the health system. The need and/or the paucity of instruments to face the “invisible unknown” enemy marks the narratives, highlighting a perceived knowledge gap as well as the inadequacy of human and material resources. The pandemic takes the form of a war fought with unequal weapons, where you can only hope to survive and to contain the losses. A similar picture was found in the analysis of the narratives of the general population where some narratives focused on the unpredictable character of the COVID-19 health emergency, experienced as a tragic, terrifying, frightening war (Venuleo et al., 2020a, b, c). However, while in that study, a representation of the pandemic crisis in terms of health emergency was opposed to a representation of the crisis as a turning point opening up a new way of managing one’s time and new awareness of the interdependence among people and countries; here, the opposite polarity identifies a more personal view of the crisis. Notably, the narratives of the health workers lying on the personal crisis polarity suggest that work pressure and distress related to the health emergency constitute only one aspect of the issue: the disruptive changes occurring in everyday life are in the foreground; the person, more than the professional role, emerges, along with their feelings of fear about potentially exposing themselves and their loved ones to infection, of bewilderment and loneliness: an image which seems a kind of response and counter-argument (Taylor, 2006) to the vision narrated and imposed by media and institutional communication, where health workers have been depicted as omnipotent “heroes” (Cassandro, 2020). It was argued that while on the one hand, the construct of hero sounds like a kind of acknowledgment of the responsiveness that health workers show in reacting to the health emergency; on the other hand, it underestimates their need to be supported both in their professional role and as human beings, and may even be dangerous when it promotes the idea that health professionals can “soldier on” at any cost and whatever the work conditions (Stokes-Parish et al., 2020; Turale et al., 2020).

It is notable that it is mainly the front-line health-workers that interpret the pandemic crisis in terms of health emergency. It is not surprising that in the daily contact with the COVID patients, daily difficulties related to the lack of human and material resources, high uncertainty about the virus, the ways to treat patients and avoid infection, along with the lack of general guidelines, the emergency became the shared frame guiding feelings and action. Spending time and “words” to focus on the disruptive change occurring in their personal life was felt to be secondary, if not also inappropriate with regard to their “mission to save lives.” Differences related to gender do not emerge. Previous studies on general samples suggest that women differed from men in the daily challenges imposed by the pandemic crisis (Hutt, 2020) and that they suffered the impact of the crisis on their well-being more than men (Connor et al., 2020). However, it is understandable that among the health workers, the shared scenario and challenges related to the medical role, to the work in hospitals, and the physical distancing from their loved ones made the experience of women and men more similar.

With regard to the second dimension (impact of the pandemic crisis), there emerge two opposite ways of depicting what the pandemic crisis produced. The powerlessness polarity foregrounds the negative impact of the pandemic on psychological health and the feeling of being helpless in face of the challenges created by the crisis, both at the level of the health system’s capacity to cope with the powerful unknown enemy, and at a personal level. On the side of the health system, several elements can be evoked to understand the sense of powerlessness (Carenzo et al., 2020): the lack of personal protective equipment (PPE), such as gloves and masks, the few places available in healthcare facilities for the number of patients in a critical condition, the insufficient human resources, a chronic shortage of healthcare workers, the overcrowding in intensive care units, with some patients dying at home while awaiting admission, and a concrete risk of being forced to treat only those with a better prognosis. On a psychological level, fear, anxiety, and feelings of loneliness are reported, consistently with the results from quantitative studies where post-traumatic stress, depression, and anxiety symptoms are cited as frequent harmful consequences of the COVID-19 pandemic on health workers (Preti et al., 2020). What the health workers’ perspective invite us to consider is how institutional responses, workplace conditions, and ordinary people’s responses (i.e., the population’s failure to comply with the proposed hygiene rules and health practices) constituted important sources of such distress, playing a significant role in constructing health workers’ vulnerability and their sense that one’s life and condition is shaped by forces outside one’s control. The notion of structural amplification can be recalled here (Ross et al., 2001): a process due to the lack of resources of the environment that undermines the personal attributes that otherwise would moderate the undesirable consequences of an objective condition or threat. In the context of SARS, Tam et al., (2004) identified inadequate psychological support, inadequate insurance/compensation, and poor sense of “team spirit” as risk factors for poor mental health; Marjanovic et al., (2007) highlighted how greater trust in equipment and infection control procedures predicted lower emotional exhaustion and a lower state of anger in nurses. Other studies have highlighted how belief that precautionary workplace measures were sufficient was associated with decreased levels of concern (Styra et al., 2008; Wu et al., 2009). In the context of the COVID-19 pandemic, Marinaci et al. (2019), based on a psychosocial perspective, analysed the relationship between emotional distress, psychosomatic symptoms and their relationship with institutional responses in a sample of Italian health-workers and found that the lower the perceived support by the institution (at the level of government, regional administration, and local health agency), the higher the probability of experiencing somatic symptoms (e.g., joint pain, headaches, stomach or bowel problems, difficulty falling asleep) as well as a perceived worsening of physical and psychological health in the previous three months.

The other polarity (Discovery of new meaning) identifies a different area of meaning, where the crisis is experienced as a rupture which, while highlighting the fragility of life and the critical impact of short-term polities, leads people to reconsider social and personal priorities: the importance of science, research, and health policies on a societal level; the value of affects in daily life; and the importance to managing one’s time better. To use an image, people’s meaning-making seems to move from the focus on loss (e.g., the dead that will never come back or the daily habits interrupted) towards a focus on what it is possible to learn from the crisis. The hope here is that of not returning to the previous state of “normality”; instead, the pandemic takes the form of a kind of semiotic antibody (Venuleo et al., 2020), a destabiliser of life and the social world, yet not fully destructive, enabling to catalyse the personal and cultural milieu’s efforts to re-consider what matters and to make the future better.

## Conclusive Remarks

The narratives of health workers report feelings of fear, anguish, and helplessness that previous research had defined and measured in terms of anxiety, depression symptoms, and psychosomatic distress. Valorising personal narratives as “data which count” allows us to exploratively examine also other kinds of problems that they bring into the foreground and, more widely, to understand how they interpret the meaning of the pandemic in their life: what the pandemic crisis represents, what kind of change it imposed, what kind of needs it brought to the fore and what should be learnt from it. Some main points are worth highlighting.

First, health workers’ narratives illuminate the risk of normalising the feelings of fear and impotence, as happens when they are seen as the direct response to an extraordinary situation. The feeling of inadequacy and powerlessness which characterise some of the narratives collected appears to be strictly intertwined with the awareness of the limited resources received to cope with the battle. In this sense, not only psychological support but strong leadership, clear direction, and more broadly, a health management designed for the global reduction of the level of uncertainty all constitute important components to prevent bewilderment and the feeling of impotence in exercising their professional role in an extraordinary circumstance like the COVID-19 pandemic. The impotence and feeling of having been abandoned related to the fact that citizens are seen to have little sense of responsibility are another important aspect. Health workers’ narratives suggest that protecting them also involves working on the construction of the symbolic resources that enable citizens to recognize that health care is a shared enterprise, and not only the responsibility of a specific professional role. The systemic nature of the healing processes emerges clearly throughout health workers’ narratives: the health of the citizens depends on the response capacities the political and health systems are able to offer individuals, just as the efforts of the health system and health workers to save citizens’ lives depends on how people feel and behave.

A further aspect that personal narratives invite us to consider is that the professional role is only one side of the health workers’ identity and medical challenges are only one area of the challenges they tried to face. Indeed, while on one hand, the narratives in some cases reflect socio-cultural membership (professional status), in other cases what emerges is the individual’s story drawing on a collective autobiography (Garro & Mattingly, 2000), foregrounding the women and men “inside the lab coat”: the suffering and loneliness related to being separated from partners and children, the sadness related to the impossibility of responding to family needs, and the lack of affection and gestures of love. Previous studies have highlighted how loneliness and lack of social support might exacerbate psychological malaise in older people, youths, women and other vulnerable groups (Ripp et al., 2020; Venuleo et al., 2020a, b, c). Health workers’ narratives invite us also to recognize the human vulnerability of a professional role that was globally presented as made up of brave heroes without fear.

Finally, processes of adjustment and personal growth emerge along with fragilities. Part of the narratives focused on how the personal and professional experience of the pandemic have significantly affected or transformed their attitude to life and their evaluation of what matters. From this perspective, the personal narratives also highlight the human effort to maintain or reconstruct a sense of self and purpose in the face of troubled circumstances.

### Limitations and Future Direction of Research

Some limitations of the present study needed be acknowledged. First, our case study is based on an Italian convenience sample; thus, the results cannot be generalised and have to be related to the specific cultural context under analysis. In other countries, other dimensions of meanings emerge to represent the pandemic crisis and its impact. Second, we did not carry out any comparative analysis with the narratives of other social groups, so we cannot establish to what extent the dimensions of meanings that we detected reflect the specific challenges faced by the health workers or the common challenges faced by Italian people in the context of political, social, and economic resources which characterised this country’s response to the pandemic crisis.

Further studies should longitudinally examine the variability of the dimension of meanings over time and their impact on psychological well-being in the medium and long term.

## Data Availability

Data are provided on request by the authors.

## Code Availability

T-Lab software—USER ID:XP6NMC39.
